# Coronary heart disease in young women: risk factors, mechanistic phenotypes, and implications for primary care

**DOI:** 10.3389/fcvm.2026.1934534

**Published:** 2026-09-21

**Authors:** Xiaoxuan Li, Xiaoran Cui

**Affiliations:** Department of Cardiology, The Second Hospital of Hebei Medical University, Shijiazhuang, China

**Keywords:** coronary heart disease, coronary microvascular dysfunction, primary care, risk stratification, sex-specific risk, spontaneous coronary artery dissection, young women

## Abstract

Coronary heart disease (CHD) in young women remains under-recognized because early risk signals are often dispersed across different clinical settings and symptoms are frequently interpreted as non-cardiac at first contact. This narrative review provides a clinically oriented synthesis that integrates conventional and female-specific cardiovascular risk factors with coronary phenotypes and practical primary-care screening, triage, referral, and longitudinal management. As a narrative review, this study employed a structured but non-systematic literature search of PubMed, CNKI, and the Chinese General Practice journal website for relevant publications available through May 2026, supplemented by manual screening of reference lists. Priority was given to recent cohort studies, systematic reviews, clinical guidelines, expert consensus statements, and representative mechanistic studies.The available literature suggests that hypertension, dysglycemia, smoking, dyslipidemia, family history, and psychosocial stress are not necessarily more severe in young women, but are more likely to be clinically underestimated. Their impact is often amplified when combined with hypertensive disorders of pregnancy, polycystic ovary syndrome, autoimmune disease, antiphospholipid syndrome, or persistent psychosocial burden. Beyond obstructive atherosclerosis, plaque erosion, coronary microvascular dysfunction, vasospasm, myocardial infarction with non-obstructive coronary arteries, and spontaneous coronary artery dissection deserve particular attention. Common primary-prevention risk scores are heavily weighted by age and generally do not fully incorporate female-specific risk-enhancing factors; they should not replace chest-pain risk stratification or mechanism-oriented evaluation in symptomatic patients.Primary care assessment should distinguish among asymptomatic individuals undergoing primary prevention, symptomatic patients presenting with chest pain, and patients with established premature CHD. In the first group, evaluation should focus on basic risk factors, including medical history, blood pressure, body mass index, lipid profile, and glycemic status. In symptomatic patients, electrocardiography and echocardiography should be selected according to the clinical context; high-sensitivity cardiac troponin should be measured when acute coronary syndrome (ACS) or acute myocardial injury is suspected, rather than used as a routine primary-care screening test. Referral for coronary computed tomography angiography or further functional/invasive assessment should be guided by clinical likelihood and the suspected mechanism. Patients with established disease require intensified secondary prevention and long-term follow-up. Recognition of CHD in young women should therefore move beyond the narrow question of visible stenosis and adopt an integrated framework that links conventional risk factors, female-specific exposures, coronary functional phenotypes, and continuity of care.

## 1 Introduction

Coronary heart disease (CHD) remains a leading cause of death and disability in women, yet the decline in overall cardiovascular mortality has not been matched by comparable gains in younger women. For the purposes of this narrative review, “young women” refers to women aged ≤55 years; the same operational threshold is used when the manuscript refers to premature CHD in women, unless an individual cited study used a different age definition. Because age thresholds vary across studies, each cited study should be interpreted according to its original eligibility criteria. Contemporary reviews and studies indicate that diagnostic delay and under-recognition remain important concerns in younger women, particularly when presentations are non-classic or non-obstructive (1–5). The challenge is not simply that events occur earlier in life. Prevailing recognition frameworks have been shaped largely by male, older, and obstructive disease patterns, with the result that many young women enter care already framed as low risk, anxiety-related, or appropriate for watchful waiting (6).

Young women rarely follow a single or uniform risk trajectory. Conventional risk factors are often present, but they may manifest as mildly elevated blood pressure, intermittent dysglycemia, atypical chest discomfort, or postpartum weight retention rather than overt metabolic disturbance. Analyses from the Framingham Study provided early epidemiological evidence that hypertension and dysglycemia attenuate the female coronary disease advantage, substantially narrowing the sex difference in CHD incidence (7). This historical observation remains relevant to contemporary accounts of premature coronary artery disease in women. At the same time, hypertensive disorders of pregnancy, polycystic ovary syndrome (PCOS), premature menopause, autoimmune disease, antiphospholipid syndrome modify how coronary risk is expressed clinically (5, 8). The American Heart Association launched Go Red for Women in 2004; over two decades, the initiative has increased public and professional awareness, supported sex-specific research, and promoted changes in prevention and clinical practice (9).

This review addresses three related questions: how conventional risk factors are amplified or clinically underestimated in young women, how female-specific exposures connect with mechanistic and phenotypic pathways of disease, and how these insights can inform practical screening, referral, and longitudinal management in primary care and multidisciplinary settings. By integrating risk recognition, coronary phenotypes, and first-contact triage within a single clinically oriented framework, this review complements previous reviews that have largely addressed sex-specific risk factors or coronary mechanisms as separate domains. The manuscript uses clinically organized, interpretive synthesis to connect different forms of evidence and their practical implications, rather than the exhaustive search, quantitative pooling, or formal evidence-grading workflow of a systematic review.

## 2 Literature search strategy

This manuscript was prepared as a narrative review. Literature searches were conducted in PubMed, CNKI, and Chinese General Practice for publications available up to May 2026.Search terms included young women, premature coronary artery disease, sex-specific cardiovascular risk, polycystic ovary syndrome, autoimmune disease, coronary microvascular dysfunction, MINOCA, and SCAD, using both Chinese and English terms and their synonyms. The search was organized around three concept groups: (1) young women/premature coronary artery disease; (2) female-specific risk exposures; and (3) INOCA, CMD, MINOCA, SCAD, and primary-care-related topics, with corresponding Chinese and English synonyms. Reference lists of included articles were also screened manually to identify additional highly relevant studies. Cohort studies, systematic reviews, clinical guidelines, and expert consensus statements published within the past five years were prioritized, while earlier landmark studies were retained for established topics when appropriate. Studies were selected according to recency, level of evidence, relevance to the three clinical questions addressed in this review, and their potential to inform risk recognition, triage, and follow-up. Because this is a narrative review, the search was intended to support a clinically oriented interpretive synthesis rather than an exhaustive systematic review. The protocol was not preregistered, screening was not performed independently by two reviewers, and no formal risk-of-bias assessment or evidence grading was undertaken.

## 3 Conventional risk factors in young women

Hypertension in young women is often more important than a single clinic reading suggests. Guidelines provide a management framework (10), and cohort data indicate that cardiovascular risk may begin to rise even when blood pressure elevation is relatively mild (11, 12). What complicates interpretation is timing. These abnormalities frequently appear around pregnancy, postpartum weight change, or periods of nonspecific symptoms, making them easy to postpone or dismiss.

If risk is judged only from one outpatient test or a brief health examination, many young women do not look conventionally high risk. The picture changes when the timeline is extended: blood pressure remains mildly elevated, postpartum weight does not return to baseline, chest tightness recurs without dramatic intensity, or a history of hypertensive pregnancy coexists with PCOS. None of these signals is striking in isolation, but together they can redirect long-term risk. Primary care misclassification often begins here.

The most common clinical miss is not extreme disease; it is the repeated deferral of mild-to-moderate abnormalities. When numbers do not appear alarming, intervention is postponed. When symptoms do not sound classic, they are attributed to anxiety, sleep disruption, or hormonal fluctuation. Over time, that delays both preventive treatment and evaluation of microvascular ischemia or pregnancy-related vascular injury. In routine community follow-up, delayed reassessment may allow persistent mild blood-pressure elevation to remain untreated for prolonged periods.

Similar issues affect dysglycemia, insulin resistance, and lipid abnormalities. Biomarker studies in women show associations between inflammatory, metabolic, and lipid markers and earlier CHD onset (13, 14), but these variables are not interchangeable and should not be interpreted outside clinical context. Low-density lipoprotein cholesterol (LDL-C) remains the main therapeutic target. Lipoprotein(a), apolipoprotein B, and triglyceride-rich remnants may refine early risk recognition, but their value is best understood as risk-enhancing information rather than a substitute for clinical history or symptom interpretation (14, 15).

Smoking, alcohol exposure, obesity, and family history are also easily underestimated in young women. Importantly, the relative cardiovascular risks associated with several conventional and behavioral factors may differ between women and men (16). Smoking has been associated with more severe coronary lesions in younger and middle-aged women with acute coronary syndromes (17), while tobacco exposure may also affect circulating sex hormone profiles in premenopausal women (18). For alcohol, the evidence should be interpreted by outcome: a large cohort of adults aged 18–65 years found that alcohol intake above guideline limits was associated with higher CHD risk (19), whereas the MESA analysis linked alcohol-consumption patterns with ectopic adiposity rather than incident CHD (20). Sex differences in alcohol pharmacokinetics and physiological responses may increase women's susceptibility to alcohol-related adverse effects at comparable intake levels (21). Obesity may confer a greater relative cardiovascular risk in women than in men (16), whereas a family history of premature coronary disease remains an important marker of genetic susceptibility and shared environmental exposure (22). A Chinese hospital-based study of premature CHD, which used a female age cutoff of ≤65 years, also identified hypertension, dyslipidemia, and diabetes as common comorbidities; its age definition is broader than the ≤55-year operational definition used in this review (23). Taken together, these findings support sex-aware assessment of conventional and behavioral risk factors without implying that every factor has the same magnitude of effect in all young women (16).

Psychosocial factors should be incorporated into the overall assessment of cardiovascular risk and symptoms. Preliminary evidence suggests an association between psychosocial stress and myocardial ischemia in young women, particularly in relation to stress-induced hemodynamic responses (24); whereas chronic stress, caregiving burden, and socioeconomic disadvantage have been associated with cardiovascular risk and adverse outcomes in women (25). Their independent causal contribution and the value of targeted interventions, however, require confirmation in large prospective studies. In clinical practice, recurrent chest pain accompanied by substantial psychological burden should not automatically be classified as noncardiac, nor should psychological factors be assumed to be the direct cause.

Pregnancy-related disorders may serve as early warning signals rather than isolated obstetric events. Hypertensive disorders of pregnancy, preeclampsia, and gestational diabetes may reflect underlying endothelial vulnerability, metabolic susceptibility, or inflammatory activation (27). International longitudinal evidence is stronger than the domestic literature in this area, and long-term Chinese follow-up data remain limited (26, 27). In practical terms, postpartum blood pressure, weight, and glycemic follow-up should be treated as part of cardiovascular prevention, not merely as the final step of obstetric care.

## 4 Female-specific risk exposures and mechanistic phenotypes

The importance of female-specific risk factors lies not only in their biological implications, but also in their timing. Hypertensive disorders of pregnancy, preeclampsia, gestational diabetes, preterm delivery, fetal growth restriction, premature menopause, PCOS, and infertility may precede overt coronary events by many years (4, 27). The evidence base, however, is not equally developed across these exposures. Associations involving hypertensive pregnancy disorders are comparatively consistent, whereas findings related to infertility treatment exposure, age at menarche, and selected endocrine markers remain more heterogeneous. Variation in cohort composition, ascertainment strategies, and duration of follow-up likely contributes to this inconsistency. Even so, these histories merit attention because they often emerge well before conventional risk scores identify a young woman as high risk.These histories can serve as risk-enhancing information in primary prevention, but they should not currently be entered directly into unvalidated female-specific risk scores, nor should they be used to exclude coronary disease in symptomatic patients; the latter should still undergo appropriate chest-pain risk stratification (27, 28).

A major diagnostic blind spot in young women arises when a reassuring angiogram is interpreted as the end of the evaluation. CMD and coronary vasospasm are important mechanisms of INOCA, whereas MINOCA is an acute working diagnosis used when the criteria for myocardial infarction are met in the absence of obstructive coronary stenosis (29, 30). In some patients, the epicardial vessels appear acceptable, yet symptoms persist, troponin rises, or stress imaging remains abnormal. This discordance illustrates the limitations of a model centered solely on luminal stenosis. The more informative question is often not whether the angiogram appears mild, but whether impaired coronary flow regulation, abnormal vasomotor function, or plaque behavior better explains the clinical presentation.

Spontaneous coronary artery dissection (SCAD), together with fibromuscular dysplasia (FMD) and pregnancy-related vascular fragility, represents a distinct pathophysiological pathway. In a subset of young women, acute coronary events are driven less by progressive plaque accumulation than by structural vulnerability of the arterial wall. This should not be regarded as an isolated curiosity. Prospective Canadian cohort data showed that most patients with SCAD were women and that many had few traditional risk factors, identifiable emotional or physical triggers, and incomplete assessment for associated arteriopathies (31, 32). When all presentations are forced into a plaque-rupture framework, both diagnosis and therapeutic decisions may be compromised.

PCOS has attracted particular attention because it may link metabolic disturbance with inflammatory and myocardial injury pathways. Some studies emphasize obesity and insulin resistance as the dominant drivers, whereas mechanistic work has described increased sympathetic activity, monocyte recruitment, and macrophage infiltration after ischemic stress (33). Study populations differ substantially in age, body composition, and background cardiometabolic risk; therefore, PCOS should not be treated as a uniform marker of coronary risk. It should instead prompt closer follow-up when exertional symptoms, dyslipidemia, elevated blood pressure, insulin resistance, or an adverse pregnancy history coexist. A 2026 global consensus renamed polycystic ovary syndrome (PCOS) as “polyendocrine metabolic ovarian syndrome” (PMOS), emphasizing the condition's broader endocrine and metabolic features (34). Because implementation of the new terminology is ongoing, PCOS remains the principal terminology used throughout this review. Exogenous and menopausal hormone therapies also require a differentiated assessment: cardiovascular effects vary with the indication, formulation and progestogen, route of administration, timing relative to menopause, and baseline thrombotic and atherosclerotic risk (35). Hormone use should therefore not be classified as uniformly protective or harmful; decisions require individualized cardiovascular and reproductive risk assessment.

Immune-mediated inflammatory disease provides a more direct bridge between systemic pathology and coronary risk. Population-based studies have linked these disorders with premature coronary artery disease (36), and lupus-focused reviews continue to support roles for chronic inflammation, endothelial dysfunction, and oxidative stress (37). Antiphospholipid syndrome is characterized by a hypercoagulable state associated with vascular thrombosis and/or pregnancy complications, and cardiovascular events can occur in affected patients (38). The literature nevertheless remains uneven. Referral-based cohorts often report stronger associations than community-based samples, and that distinction matters when specialist evidence is translated into frontline risk assessment.

Plaque erosion is another phenotype that deserves closer attention in younger women presenting with acute coronary syndromes. Recent reviews indicate that plaque erosion accounts for a substantial minority of ACS and is reported more often in younger patients and women than plaque rupture. Pathologically, plaque erosion is characterized by endothelial denudation with superimposed thrombus rather than rupture of a large lipid-rich necrotic core (39). Rather than the classic picture of a large ruptured lipid core, the lesion more often involves endothelial denudation with superimposed thrombus. That distinction may be easy to flatten in broad review narratives, but it has practical relevance. It cautions against assuming that every acute event in a young woman follows the same sequence typically described in older men with advanced obstructive disease.

INOCA and MINOCA should not be treated as diagnostic labels at the same clinical level or stage. INOCA primarily describes chronic or stable myocardial ischemia in the setting of nonobstructive coronary arteries, commonly driven by CMD and epicardial or microvascular spasm (29, 30). Microvascular angina is common among patients with stable symptoms and no obstructive coronary artery disease, although reported prevalence varies substantially with the diagnostic definition and testing strategy (41). MINOCA, by contrast, is a working diagnosis applied when criteria for acute myocardial infarction are met and coronary stenosis is <50%, and it requires further etiologic evaluation (30, 40). SCAD, plaque rupture/erosion, coronary spasm, and embolism may account for the coronary cause in some MINOCA cases; however, SCAD is itself a nonatherosclerotic disease of the arterial wall and should not be used interchangeably with INOCA or CMD (40, 43). The VIRGO cohort underscored this heterogeneity: young patients with MINOCA were more often women, displayed mixed mechanisms, and could not simply be regarded as a low-risk subgroup (42). This also helps explain why guidelines and consensus statements do not fully converge. Differences in study populations, diagnostic definitions, availability of intracoronary imaging or coronary functional testing, and follow-up methods may contribute to variability in reported prognosis and management approaches across MINOCA and SCAD studies (40, 43). In other words, some apparent disagreement in the literature reflects differences in case mix and diagnostic depth rather than a true therapeutic contradiction.

INOCA, CMD, MINOCA, and SCAD should be understood according to their distinct clinical levels and mechanisms. CMD is an important mechanistic phenotype of INOCA, and vasospastic angina may arise from either epicardial or microvascular spasm; the two can coexist (29, 30). MINOCA is a working diagnosis in acute myocardial infarction, with potential causes including plaque rupture/erosion, severe coronary spasm, SCAD, and coronary embolism; further testing is required to establish the final diagnosis (30, 40). SCAD is a nonatherosclerotic arterial-wall disorder characterized by intramural hematoma and/or an intimal tear that compresses the true lumen. It occurs predominantly in women and is associated with FMD and certain pregnancy-related settings (31, 32, 43). Clinical pathways should therefore first distinguish stable ischemia from acute myocardial infarction and then select functional testing, imaging, and treatment according to the suspected mechanism rather than grouping these terms as equivalent diagnoses.

Taken together, female-specific exposures and mechanistic pathways tend to cluster into three broad clinical patterns: earlier obstructive atherosclerosis, functional ischemic syndromes dominated by endothelial or microvascular dysfunction, and arterial wall fragility or prothrombotic states represented by SCAD or thrombotic events. These categories are clinically useful, although they are not entirely discrete in individual patients.

Socioeconomic and psychosocial vulnerability represent important influences outside the coronary image and are associated with cardiovascular risk and care processes in women (25). Education, access to healthcare, caregiving burden, and social support may influence when patients seek care, how symptoms are interpreted, and whether follow-up is sustained. Broader human environmental exposures have also been linked to cardiometabolic risk through multi-omic pathways (44). Domestic evidence on delayed presentation, barriers to recognition, and breakdowns in continuity of care among young women remains limited, and this remains one of the most practical gaps in the current literature.

## 5 Implications for primary care screening and triage

To avoid conflating different clinical goals, primary care assessment should distinguish among three groups: asymptomatic individuals undergoing primary prevention, symptomatic patients presenting with chest pain, and patients with established premature CHD. For symptomatic patients, the key clinical question is not whether all should undergo extensive cardiovascular testing, but whether primary care can identify those who should remain on a coronary evaluation pathway rather than experience diagnostic delay after repeated reassurance. For asymptomatic individuals, the priority is to identify established and risk-enhancing exposures. In addition to family history, smoking, hypertension, diabetes, dyslipidemia, and obesity, clinicians should ask about adverse pregnancy outcomes, premature menopause, PCOS and other relevant reproductive history, chronic inflammatory or autoimmune disease, antiphospholipid syndrome, psychosocial stress, hormonal contraception or hormone therapy, and stimulant or other drug exposure when clinically relevant (4, 16, 27, 28, 35, 45, 46). Common 10-year cardiovascular risk scores are intended primarily for primary prevention. Because younger age may result in a low estimated absolute short-term risk and female-specific risk-enhancing factors are often not fully incorporated, a low risk score should not replace clinical judgment based on reproductive history, autoimmune disease, and other risk-enhancing factors (4, 27). Symptomatic patients should instead be evaluated using the appropriate chest pain or acute coronary syndrome (ACS) risk pathway rather than a primary-prevention risk score (47). Current nationwide data indicate that combined hormonal contraception is associated with a small but measurable increase in the risks of ischemic stroke and myocardial infarction, with the absolute risk varying according to the contraceptive formulation and baseline risk (45). Drug misuse, particularly involving sympathomimetic agents, is associated with vascular injury and premature cardiovascular events and may precipitate coronary vasospasm or ischemia (46). These risk factors are frequently omitted from routine history taking, particularly when symptoms are considered atypical. For patients with established premature CHD, the focus should shift toward the intensity of secondary prevention and individualized treatment adjustment.

A clearer distinction should be made between primary and specialist care. In asymptomatic individuals undergoing primary prevention, baseline assessment should focus on medical history, blood pressure and weight measures, glucose, lipids, and renal function when indicated. In symptomatic patients, a 12-lead ECG is a common initial test; high-sensitivity troponin should be measured through an acute chest-pain pathway when acute myocardial injury or ACS is suspectedand should not be used as a routine screening test in otherwise stable primary-care patients. Echocardiography should be used when structural heart disease, cardiac dysfunction, or other clinical indications are present. CCTA and noninvasive functional imaging should be selected according to clinical likelihood and symptom characteristics. Invasive coronary angiography, intracoronary imaging, microvascular function testing, and pharmacologic provocation testing are generally specialist-level investigations rather than routine primary care screening tools. This stepwise approach is consistent with both the 2021 AHA/ACC chest pain guideline, which cautions against routine urgent testing in low-risk patients, and the 2024 ESC chronic coronary syndrome guideline, which expands attention to ANOCA/INOCA while still emphasizing pre-test likelihood and selective downstream investigation (47, 48). The two documents are broadly aligned on triage, although their emphasis differs: the AHA/ACC pathway is anchored in front-door chest pain evaluation, whereas the ESC guideline goes further in the structured assessment of non-obstructive and functional syndromes.

Table 1 shows a pragmatic screening and referral framework for first-contact care.

**Table 1:** Primary care screening and referral priorities.

Scenario	Initial assessment feasible in primary care	Key clues suggesting higher risk	Suggested action
Asymptomatic but with risk exposure	Blood pressure, body mass index, waist circumference, fasting glucose/HbA1c, lipid profile, renal function; ask about premature family history, pregnancy complications, PCOS, autoimmune disease, smoking, and sleep	Prior preeclampsia/gestational diabetes, premature family history of CHD, markedly elevated LDL-C or Lp(a), clustering of risk factors	Initiate lifestyle intervention and risk-factor management; schedule reassessment according to baseline risk, the degree of abnormality, and treatment-adjustment needs. Consider cardiology referral when multiple risk-enhancing factors are present or management is difficult.
Atypical chest pain, exertional dyspnea, recurrent palpitations or fatigue	12-lead ECG; high-sensitivity troponin when ACS or acute myocardial injury is suspected (not as a routine primary-care screening test); echocardiography when clinically indicated; blood pressure and heart rate assessment; concurrent assessment of anxiety, depression, and sleep	Exertional symptoms, ischemic ECG changes at rest, abnormal troponin, repeated healthcare visits without explanation	Determine the urgency of referral according to persistent/progressive symptoms, ECG or troponin abnormalities, and structured risk assessment; refer to the appropriate specialist pathway when CCTA, functional imaging, or coronary function testing is required.
Persistent acute chest pain or suspected acute coronary syndrome	Immediate ECG, vital signs, and high-sensitivity troponin testing when ACS/acute myocardial injury is suspected	persistent or progressive chest pain at rest, hemodynamic instability, new ischemic ECG changes, syncope, or new signs of heart failure	Emergency transfer and management along acute coronary syndrome pathways
Autoimmune disease, antiphospholipid syndrome, or suspected SCAD/FMD	Ask about thrombosis, pregnancy history, recurrent miscarriage, migraine, and vascular abnormalities in other arterial beds	Young age, few conventional risk factors, peripartum onset, a history of thrombosis or recurrent pregnancy loss, mismatch between angiographic findings and symptoms	Seek early specialist input; avoid reflex attribution to anxiety or functional complaints

CHD, coronary heart disease; ECG, electrocardiography; HbA1c, glycated hemoglobin; LDL-C, low-density lipoprotein cholesterol; Lp(a), lipoprotein(a); PCOS, polycystic ovary syndrome; SCAD, spontaneous coronary artery dissection; FMD, fibromuscular dysplasia. Source: compiled by the authors based on Refs (6, 26–28, 47, 48, 50).

A diagnostic pathway can be organized around two complementary tracks—anatomic disease and functional disturbance—but test selection should be guided by symptoms, clinical likelihood, and prior results. In symptomatic patients with an intermediate or higher likelihood of obstructive coronary disease, coronary CTA or functional imaging may be selected according to guidelines and local availability; invasive coronary angiography and OCT/IVUS are reserved for specific high-risk or invasive-evaluation contexts and should not be routine primary-care tests. Patients with persistent or recurrent symptoms and documented nonobstructive coronary arteries should undergo specialist consideration of ANOCA/INOCA and its mechanistic endotypes, whereas patients meeting myocardial-infarction criteria with nonobstructive coronary arteries should proceed through a MINOCA etiologic work-up. Recent guidelines have further structured these distinct pathways. The 2024 ESC guideline explicitly broadens the diagnostic frame for ANOCA/INOCA, whereas the 2021 AHA/ACC chest pain guideline is particularly strong on risk stratification and avoidance of unnecessary testing in low-risk presentations (47, 48). The difference is less a true conflict than a difference in clinical entry point, but it helps explain why young women may still undergo multiple investigations without receiving a coherent diagnosis.

## 6 Phenotype-informed management and longitudinal care

Management of confirmed obstructive atherosclerotic CHD in young women still rests on the standard pillars of risk-factor control, antithrombotic therapy, intensive lipid lowering, and revascularization when indicated. The distinction lies in the details of longitudinal care, including pregnancy planning, the effect of menstrual bleeding on antithrombotic tolerance, and when to adjust statins or angiotensin-converting enzyme inhibitor/angiotensin receptor blocker (ACEI/ARB) therapy before conception. Primary care is not expected to replace specialist decision making in this setting, but it remains central to medication adherence, serial risk reassessment, and safety monitoring.

INOCA should be managed according to its specific endotype rather than simply as ‘no stenosis.’ For CMD or vasospasm, lifestyle measures and individualized antianginal and risk-factor treatment can be guided by symptoms, risk factors, and functional findings; vasospastic phenotypes are generally managed with calcium-channel blockers and avoidance of triggers such as smoking (29, 30, 48). MINOCA is different: it is a working diagnosis in acute myocardial infarction, and treatment should be directed by the underlying cause once further evaluation identifies plaque rupture/erosion, spasm, SCAD, embolism, or another mechanism, rather than applying a uniform regimen before the cause is known (40, 42). The use of advanced functional or imaging tests should therefore be determined by the clinical context, persistent symptoms, and the suspected mechanism.

Stable SCAD is usually managed conservatively, with close follow-up and avoidance of unnecessary PCI unless ongoing ischemia, hemodynamic instability, or high-risk anatomy is present (43, 49). The Canadian SCAD cohorts support that cautious approach. Early invasive management may be technically challenging, and recurrent events or persistent symptoms remain relevant during follow-up (31, 32). Screening for FMD and other systemic arteriopathies should therefore be incorporated into longitudinal care rather than treated as an afterthought. When autoimmune disease, antiphospholipid syndrome, PCOS, or pregnancy-related risk factors coexist, coordination among cardiology, obstetrics and gynecology, endocrinology, and rheumatology becomes part of the treatment strategy itself.

Table 2 outlines phenotype-oriented management and follow-up priorities.

**Table 2:** Phenotype-oriented management and follow-up priorities.

Phenotype	Key priorities feasible in primary care/general practice	Specialist collaboration points	Reproductive considerations
Obstructive atherosclerotic CHD	Control blood pressure, lipids, glucose, weight, and smoking; reinforce adherence and scheduled follow-up	Assess the need for CCTA/invasive angiography and revascularization according to symptoms, ischemic risk, and prior anatomic information; invasive testing should be limited to appropriate indications.	Review statins, ACEI/ARB therapy, and antithrombotic regimens before planned pregnancy
INOCA (including CMD/coronary vasospasm endotypes)	Symptom diary, sleep and stress intervention, exercise prescription, monitoring of medication response	In patients with persistent symptoms and no obstructive CAD, use perfusion imaging or coronary function testing as guided by specialist assessment to distinguish CMD from epicardial or microvascular spasm and other endotypes.	Assess the suitability of beta-blockers or calcium-channel blockers before pregnancy
MINOCA/SCAD (specific etiologies)	Recognize recurrent chest pain and new warning symptoms of ischemia and reinforce follow-up education; activity during SCAD recovery should be individualized, with unnecessary extreme physical exertion avoided.	MINOCA requires further etiologic clarification (including CMR and intracoronary imaging/function testing when appropriate). Stable SCAD is generally managed conservatively, with specialist assessment of FMD screening, antithrombotic therapy, and imaging follow-up.	Joint cardiology-obstetric risk assessment before future pregnancy;for MINOCA, assess pregnancy-related risk according to the established etiology
PCOS/pregnancy complications/autoimmune-related high-risk status	Postpartum and long-term metabolic management; structured documentation of pregnancy, miscarriage, and autoimmune history	Build shared care with obstetrics and gynecology, endocrinology, and rheumatology	Prioritize blood pressure, glucose, and thrombotic risk control around conception and pregnancy

ACEI, angiotensin-converting enzyme inhibitor; ARB, angiotensin receptor blocker; CHD, coronary heart disease; CMD, coronary microvascular dysfunction; CTA, coronary computed tomography angiography; FMD, fibromuscular dysplasia; INOCA, ischemia with non-obstructive coronary arteries; MINOCA, myocardial infarction with non-obstructive coronary arteries; PCI, percutaneous coronary intervention; PCOS, polycystic ovary syndrome; SCAD, spontaneous coronary artery dissection. Source: compiled by the authors based on Refs (6, 26, 27, 29–32, 40–43, 49).

In practical terms, the complexity of CHD in young women becomes clinically useful only when it is translated into concrete decisions: which questions should be asked at first contact, which findings should lower the threshold for referral, and who requires postpartum or preconception follow-up rather than episodic reassurance. From a healthcare-delivery perspective, earlier recognition and more targeted triage could potentially reduce repeated low-yield visits and unnecessary testing while directing specialist resources toward patients most likely to benefit; however, these system-level effects require prospective evaluation.

## 7 Limitations

This review has several limitations. First, it was designed as a narrative review rather than a formal systematic review. Study selection was non-systematic and retained an unavoidable interpretive component, creating a risk of selection bias; we did not perform duplicate screening, a formal evidence-quality assessment, or a risk-of-bias evaluation of included studies. Second, definitions of “young women,” “premature CHD,” and “early-onset CHD” vary across the literature. The cited cohorts, mechanistic studies, and guideline-based recommendations also used different inclusion criteria, diagnostic pathways, and follow-up durations, limiting direct comparison and synthesis. Third, long-term data in young women remain uneven across regions, particularly for functional coronary syndromes and female-specific exposures. Outcome data for pregnancy complications, PCOS, infertility treatments, hormonal contraception, and menopausal hormone therapy are limited or heterogeneous. Fourth, prioritizing recent, clinically relevant, and accessible publications may have underrepresented null findings or literature outside the searched databases. These constraints limit causal inference and generalizability and should be considered when applying this synthesis to clinical decision making, primary care triage, and healthcare planning.

## 8 Conclusions

The distinctiveness of CHD in young women does not lie in a wholly separate disease framework, but in the earlier, more diffuse, and more easily misinterpreted way that familiar risks are expressed. Conventional metabolic abnormalities, reproductive endocrine exposures, immune-inflammatory conditions, psychosocial stress, and functional ischemic phenotypes interact to produce clinical pathways that are often not captured by standard risk models. A healthcare-oriented response should begin with more careful first-contact history taking, more disciplined risk interpretation in primary care, phenotype-informed referral, and sustained multidisciplinary follow-up. Earlier recognition and better triage may improve the efficiency of diagnostic pathways, but effects on emergency presentations, repeated testing, specialist-resource use, and healthcare costs should be confirmed prospectively. Future work will be most valuable if it clarifies which history elements best identify high-risk young women in routine care, how domestic cohorts link female-specific exposures to long-term coronary outcomes, and how phenotype-specific pathways can reduce delayed diagnosis without increasing unnecessary testing or long-term healthcare costs.
